# Trends in Prescription Medication Use for Depression Symptoms: An Analysis of National Health Interview Survey (NHIS) Data From 2019 to 2023

**DOI:** 10.7759/cureus.84944

**Published:** 2025-05-28

**Authors:** Feyisayo O Oguntuase, Okelue E Okobi, Oluseyi O Olawale, Osatohanmwen Irorere, Oluwatayo A Dare, Nnenna B Emejuru, Roseline Igbadumhe, Oyindamola D Duyilemi, Saliu A Shittu, Erhieyovbe Emore

**Affiliations:** 1 General Medicine, National Pirogov Memorial Medical University, Vinnitsa, UKR; 2 Family Medicine, IMG Research Academy and Consulting, Homestead, USA; 3 Family Medicine, Larkin Community Hospital Palm Springs Campus, Miami, USA; 4 Family Medicine, Lakeside Medical Center, Belle Glade, USA; 5 Family and Community Medicine, Walden University, Minneapolis, USA; 6 Psychiatry, Neurology, Internal Medicine, Caribbean Medical University, Houston, USA; 7 Psychiatry, Foothills Medical Clinic, Calgary, CAN; 8 Psychiatry and Behavioral Sciences, College of Medicine, IMO State University, Orlu, NGA; 9 Psychology, Alaska Native Medical Center, Anchorage, USA; 10 Psychiatry, All Saints University, School of Medicine, Atlanta, USA; 11 Nephrology, Oasis Kidney Care Center, Houston, USA; 12 Anatomy, Delta State University, Abraka, NGA

**Keywords:** antidepressant use, depression, mental health, nhis, social vulnerability, sociodemographics, trends

## Abstract

Background: Depression is a major public health concern, and antidepressant medication is commonly prescribed for its management. Understanding trends in antidepressant use across socio-demographic groups is crucial for targeted interventions.

Objective: To analyze trends in antidepressant medication use between 2019 and 2023 using data from the National Health Interview Survey (NHIS), focusing on demographic factors such as gender, age, race, and social vulnerability.

Method: Data from the NHIS (2019-2023) were analyzed to assess trends in antidepressant use by sociodemographic variables. Descriptive statistics and trends were evaluated using prevalence estimates with confidence intervals.

Result: Overall, antidepressant use increased from 9.8% (95% CI: 9.4-10.3) in 2019 to 11.4% (95% CI: 11.0-11.9) in 2023 (*p*-trend <0.001). The most notable increases were observed among females (13.3% in 2019 to 15.3% in 2023; *p*-trend <0.05), individuals aged 45-64 years (*p*-trend <0.05), and those with higher social vulnerability (*p*-trend =0.004). Racial disparities persisted, with White individuals showing the highest use of antidepressants (11.2% in 2019 to 13.2% in 2023; *p*-trend <0.05). Use was significantly higher among those with disabilities compared to those without (27.7% vs. 8.1% in 2019; 28.2% vs. 9.7% in 2023; *p* <0.001).

Conclusion: The study reveals a steady increase in antidepressant use from 2019 to 2023, particularly among females, older adults, and individuals with higher social vulnerability or disabilities. Racial disparities in antidepressant use persist, with White individuals showing the highest prevalence. These findings highlight the ongoing need for targeted mental health interventions, especially for vulnerable groups, and underscore the importance of addressing disparities in access to mental health care. Future research should focus on the factors driving these trends and their implications for public health.

## Introduction

Depression, a deeply pervasive mental health disorder, casts a shadow over daily life, diminishing joy, motivation, concentration, appetite, and sleep [1-3]. Major symptoms include persistent depressed mood, anhedonia, significant weight change, insomnia or hypersomnia, psychomotor agitation or retardation, fatigue, feelings of worthlessness, impaired concentration, and recurrent thoughts of death [1-3]. Beyond its toll, it strains healthcare systems and economies [1-3]. In recent years, growing awareness, evolving policies, such as the Mental Health Parity and Addiction Equity Act and expanded telehealth reimbursement, and shifting perceptions have reshaped how we approach its treatment [3-4]. Prescription medication use, once stigmatized and static, now reflects these changing tides, mirroring broader shifts in mental health care. Analyzing these patterns offers a vital window into how society is responding to the rising demand for effective, accessible depression management. Understanding these shifts is key to shaping future interventions and improving patient outcomes [5].

Depression affects millions of individuals globally, with the World Health Organization ranking it as a leading cause of disability worldwide [2]. In the United States alone, data from the National Institute of Mental Health indicate that approximately 21 million adults experienced at least one major depressive episode in 2021, representing 8.3% of all adults, with higher rates in females (10.3%), young adults aged 18-25 (18.6%), and multiracial individuals (13.9%). Males had a lower prevalence of 6.2% [6]. The prevalence is notably higher among women, young adults, and individuals with chronic health conditions or socioeconomic disadvantages. Over the last decade, societal stressors such as economic instability, social isolation, and, more recently, the COVID-19 pandemic, have further exacerbated the burden of depressive disorders. These trends underscore the growing importance of effective treatment strategies, including pharmacological interventions [4,7].

Depression is a multifactorial psychiatric disorder characterized by complex interactions between neurobiological, genetic, and environmental factors [1]. Pathophysiologically, it involves significant dysregulation in monoaminergic neurotransmitter systems, particularly serotonin, norepinephrine, and dopamine, which are crucial in mood regulation [8]. Hyperactivity of the hypothalamic-pituitary-adrenal (HPA) axis leads to elevated cortisol levels, contributing to impaired stress response and hippocampal atrophy (i.e., shrinkage of the hippocampus, a brain region critical for memory and emotion regulation) [1,9]. Neuroinflammatory processes marked by elevated pro-inflammatory cytokines also play a role, disrupting synaptic transmission and neuroplasticity (i.e., the brain’s ability to form and reorganize synaptic connections) [10]. Structural and functional abnormalities in key brain regions, such as the prefrontal cortex, hippocampus, and amygdala, have been consistently observed through neuroimaging [11]. These changes inform the therapeutic mechanisms of antidepressants that aim to correct neurotransmitter imbalances and restore neurocircuit integrity [11].

This study utilizes publicly available data from the National Health Interview Survey (NHIS) for the years 2019 through 2023 [12]. The NHIS, administered by the Centers for Disease Control and Prevention’s National Center for Health Statistics (NCHS), is one of the principal sources of information on the health of the U.S. population. It employs a cross-sectional household interview design and collects data through personal interviews conducted annually. The survey includes modules on health conditions, healthcare access, and prescription medication use, enabling an in-depth examination of trends in depression treatment over time. By analyzing this robust dataset, researchers can identify demographic and temporal variations in prescription patterns for depression symptoms.

The primary objective of this study is to analyze national trends in the use of prescription medications for the treatment of depression symptoms among U.S. adults between 2019 and 2023. Specifically, it aims to (1) quantify changes in antidepressant use over time, and (2) examine differences across demographic subgroups such as age, gender, race/ethnicity. The findings from this analysis can provide critical insights for clinicians, policymakers, and mental health advocates seeking to optimize depression management strategies in the evolving healthcare landscape.

## Materials and methods

Data source and study design

This study employed a descriptive secondary data analysis of cross-sectional NHIS data spanning 2019 through 2023. The NHIS, administered by the Centers for Disease Control and Prevention (CDC)’s National Center for Health Statistics (NCHS), is an annual, nationally representative household survey of the civilian, non-institutionalized U.S. population. It uses a multistage probability sampling method to ensure representativeness across states and demographic strata. Public-use datasets for each survey year were downloaded from the CDC NHIS repository, de-identified, and combined into a single analytic file.

Study participants and questionnaires

Participants were adults aged ≥18 years who completed the NHIS Sample Adult Core questionnaire in any year from 2019 to 2023 (total invited sample across five cycles: n=155,000). The NHIS employs stratified, multistage probability sampling to select households, then one adult per household for the Sample Adult Core. Respondents included those who (1) answered “yes” to standardized screening questions on feelings of depression, sadness, or loss of interest; and (2) self‐reported use of prescription medication for these symptoms. This defined our primary analytic sample (n=14,820, 9.6% of total). Respondents missing data on key variables (depression screening items or medication use) were excluded (n=1,230). The Sample Adult Core questionnaire comprises modules on demographics, health conditions, healthcare access, mental health, and prescription medication use. Depressive symptoms were assessed via standardized questions and the Kessler Psychological Distress Scale (K6), a validated six‐item instrument (items scored 0 “none of the time” to 4 “all of the time,” total score range 0-24; scores ≥13 indicate serious psychological distress).

Data collection and quality assurance

NHIS data were collected via structured face-to-face interviews, supplemented in some years (2020 and 2021) by telephone follow-ups. Interviewers were trained extensively to maintain consistency and reduce interviewer bias. To ensure quality and reliability, data underwent multiple rounds of verification and validation by NCHS. Public-use datasets were cleaned and de-identified prior to release. For this study, data were downloaded from the CDC’s official NHIS repository and combined across five years, with necessary harmonization of variable names and response formats to account for annual changes in survey design.

Variables of interest

The primary outcome variable was the self-reported use of prescription medication for depression symptoms. Depressive symptoms were assessed via two approaches: (1) affirmative responses to NHIS items asking whether, in the past 30 days, respondents “felt depressed,” “felt sad,” or “lost interest in most activities;” and (2) the Kessler Psychological Distress Scale (K6), a six-item instrument scored 0 (“none of the time”) to 4 (“all of the time”) per item, with total scores ≥13 indicating serious psychological distress. Key independent variables included demographic characteristics such as age groups (categorized into 18-44, 45-64, 65-74, and ≥75 years), gender (Male and Female), race/ethnicity (American Indian or Alaska Native only, Asian only, Black only, White only), poverty level (Federal Poverty Level [FPL]: less than 100% FPL, 100% to less than 200% FPL, and ≥200% FPL), and insurance status (Private, Medicaid or other public, other coverage, and uninsured). Secondary variables encompass lifestyle factors such as social vulnerability, assessed using the CDC Social Vulnerability Index (SVI) percentile ranks, categorized into little to no (0-25th percentile), low (26-50th), medium (51-75th), and high (76-100th) social vulnerability, and disability (With and Without). These variables were selected based on relevance to depression treatment patterns and their availability across all five survey cycles.

Data analysis and statistical methods

Descriptive statistics were used to summarize participant characteristics and annual trends in prescription medication use. Chi-square tests and t-tests compare differences across variables, and the p-value is calculated by Chi-square tests (p<0.05). Subgroup analyses explored variations by age, gender, race/ethnicity, and insurance coverage. All analyses incorporated sampling weights and complex survey design variables using SPSS version 30 to ensure nationally representative estimates and accurate variance calculations.

Ethical considerations

This study was based on publicly available, de-identified NHIS data, which does not require institutional review board (IRB) approval. However, the study adhered to ethical principles for secondary data analysis, ensuring data confidentiality and appropriate use of health information. All analyses were conducted in compliance with CDC and NCHS data use policies.

## Results

The result presented the trends in antidepressant medication use among U.S. adults from 2019 to 2023, stratified by demographic and socioeconomic variables, highlighting significant patterns and disparities. Table 1 presents annual prevalence rates with 95% confidence intervals for depression-related prescription medication use across gender, age, race, veteran status, disability, poverty level, insurance type, and social vulnerability from 2019 to 2023. Table 1 below indicates the trends in prescription medication use for depression symptoms among US adults by demographic and socioeconomic characteristics between 2019 and 2023.

**Table 1:** Demographic and socioeconomic characteristics (2019–2023) Trends in prescription medication use for depression symptoms among U.S. adults. FPL: Federal poverty level.

Category	Year	2019	2020	2021	2022	2023	P value
Overall data	Overall	9.8 (9.4-10.3)	10.3 (9.8-10.8)	10.6 (10.2-11.0)	11.5 (11.0-12.0)	11.4 (11.0-11.9)	<0.001
Based on Gender	Female	13.3 (12.7-14.0)	13.9 (13.2-14.6)	13.9 (13.3-14.6)	15.2 (14.5-15.9)	15.3 (14.7-16.0)	<0.05
	Male	6.1 (5.7-6.6)	6.4 (5.9-6.9)	7.0 (6.5-7.5)	7.6 (7.1-8.2)	7.3 (6.8-7.9)	
Based on age	18-44 years	7.6 (7.1-8.2)	8.7 (8.0-9.4)	9.6 (9.0-10.3)	10.1 (9.4-10.9)	10.7 (10.1-11.4)	<0.05
	45-64 years	11.9 (11.1-12.7)	12.1 (11.3-12.9)	12.0 (11.3-12.7)	13.0 (12.2-13.8)	12.1 (11.4-12.9)	
	65-74 years	12.6 (11.6-13.7)	12.3 (11.3-13.4)	11.8 (10.8-12.9)	13.0 (12.0-14.1)	12.4 (11.4-13.5)	
	75 years and over	9.8 (8.7-11.0)	9.1 (8.0-10.2)	8.9 (7.9-9.9)	11.1 (9.9-12.4)	11.3 (10.1-12.6)	
Based on Veteran status	Veteran	10.1 (8.9-11.5)	10.9 (9.5-12.6)	10.0 (8.8-11.4)	11.4 (10.0-12.9)	11.0 (9.6-12.6)	0.0345
	Non-veteran	9.8 (9.4-10.3)	10.3 (9.8-10.8)	10.6 (10.2-11.1)	11.7 (11.2-12.2)	11.5 (11.0-12.0)	
Based on race	American Indian or Alaska Native only	7.3 (3.7-12.6)	10.0 (6.3-14.8)	9.5 (5.2-15.4)	10.9 (7.2-15.7)	8.2 (5.3-12.1)	<0.05
	Asian only	3.3 (2.3-4.6)	2.9 (2.0-4.0)	3.2 (2.3-4.4)	3.0 (2.1-4.1)	3.5 (2.5-4.6)	
	Black only	7.1 (6.2-8.2)	6.9 (5.8-8.1)	6.4 (5.4-7.4)	7.2 (6.2-8.3)	7.7 (6.6-8.8)	
	White only	11.2 (10.7-11.7)	12.1 (11.5-12.6)	12.4 (11.9-12.9)	13.5 (13.0-14.1)	13.2 (12.7-13.8)	
Based on social vulnerability	Little to no social vulnerability	10.0 (9.0-11.0)	11.3 (10.4-12.3)	12.0 (10.9-13.2)	13.0 (11.7-14.5)	12.4 (11.2-13.6)	0.004465
	Low social vulnerability	9.8 (9.0-10.7)	10.8 (9.8-11.8)	10.7 (9.9-11.4)	12.5 (11.5-13.6)	12.3 (11.3-13.2)	
	Medium social vulnerability	10.1 (9.4-10.9)	10.5 (9.7-11.4)	11.0 (10.3-11.8)	12.4 (11.5-13.4)	12.3 (11.5-13.2)	
	High social vulnerability	9.4 (8.5-10.3)	8.6 (7.7-9.6)	8.7 (7.9-9.6)	9.4 (8.7-10.2)	9.8 (9.1-10.5)	
Based on Disability	With disability	27.7 (25.8-29.6)	25.7 (23.7-27.7)	27.5 (25.5-29.6)	29.6 (27.5-31.7)	28.2 (26.3-30.1)	<0.05
	Without disability	8.1 (7.7-8.5)	8.8 (8.4-9.3)	9.0 (8.6-9.4)	9.7 (9.2-10.1)	9.7 (9.3-10.2)	
Based on Poverty level	Less than 100% FPL	13.9 (12.4-15.5)	14.7 (13.0-16.5)	14.1 (12.6-15.8)	16.0 (14.3-17.7)	14.7 (13.3-16.3)	<0.05
	100% to less than 200% FPL	11.7 (10.7-12.8)	11.5 (10.4-12.7)	12.1 (11.0-13.2)	13.1 (12.0-14.3)	13.2 (12.0-14.4)	
	200% and greater FPL	8.7 (8.3-9.1)	9.4 (8.9-9.9)	9.8 (9.3-10.2)	10.5 (10.0-11.1)	10.5 (10.0-11.0)	
Based on insurance	Private	8.4 (7.9-8.9)	8.9 (8.3-9.5)	9.6 (9.1-10.2)	10.3 (9.6-10.9)	10.6 (10.0-11.3)	<0.05
	Medicaid or other public	15.9 (14.3-17.5)	16.9 (15.0-18.9)	17.3 (15.7-19.1)	18.1 (16.4-19.8)	16.7 (15.2-18.3)	
	Other coverage	19.6 (16.9-22.6)	21.5 (18.1-25.2)	22.1 (18.9-25.6)	22.2 (19.1-25.5)	21.3 (18.1-24.7)	
	Uninsured	4.7 (3.8-5.6)	5.1 (4.0-6.3)	4.6 (3.7-5.6)	4.2 (3.3-5.3)	4.0 (3.1-5.0)	

Overall data

From 2019 to 2023, there was a steady increase in the overall use of antidepressant medications among the population. In 2019, the prevalence was 9.8% (95%CI: 9.4-10.3), which climbed gradually over the years, reaching 11.5% (95%CI: 11.0-12.0) in 2022, and slightly decreased to 11.4% (95%CI: 11.0-11.9) in 2023 (p-value <0.001) (Table 1). This consistent upward trend suggests an increasing reliance on or improved access to mental health care and antidepressant prescriptions during this period.

Based on gender

**Figure 1:**
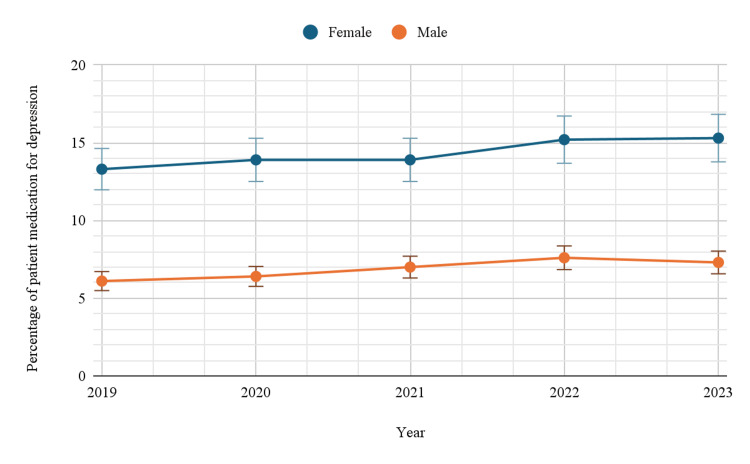
Trends in depression-related prescription medication use by gender

Gender-based analysis revealed a significant disparity in antidepressant usage, with women consistently demonstrating higher rates than men (p < 0.05). In 2019, 13.3% (95%CI: 12.7-14.0) of females reported using antidepressants, compared to only 6.1% (95%CI: 5.7-6.6) of males. This gender gap persisted and widened slightly over time, culminating in 2023 with a prevalence of 15.3% (95%CI: 14.7-16.0) among females, while males recorded a much lower prevalence of 7.3% (95%CI: 6.8-7.9) (Table 1). A significant difference was observed in trends over time based on gender, with a p-value of <0.05. Figure 1 below indicates the trends in depression-related prescription medication use by gender.

Based on age groups

**Figure 2:**
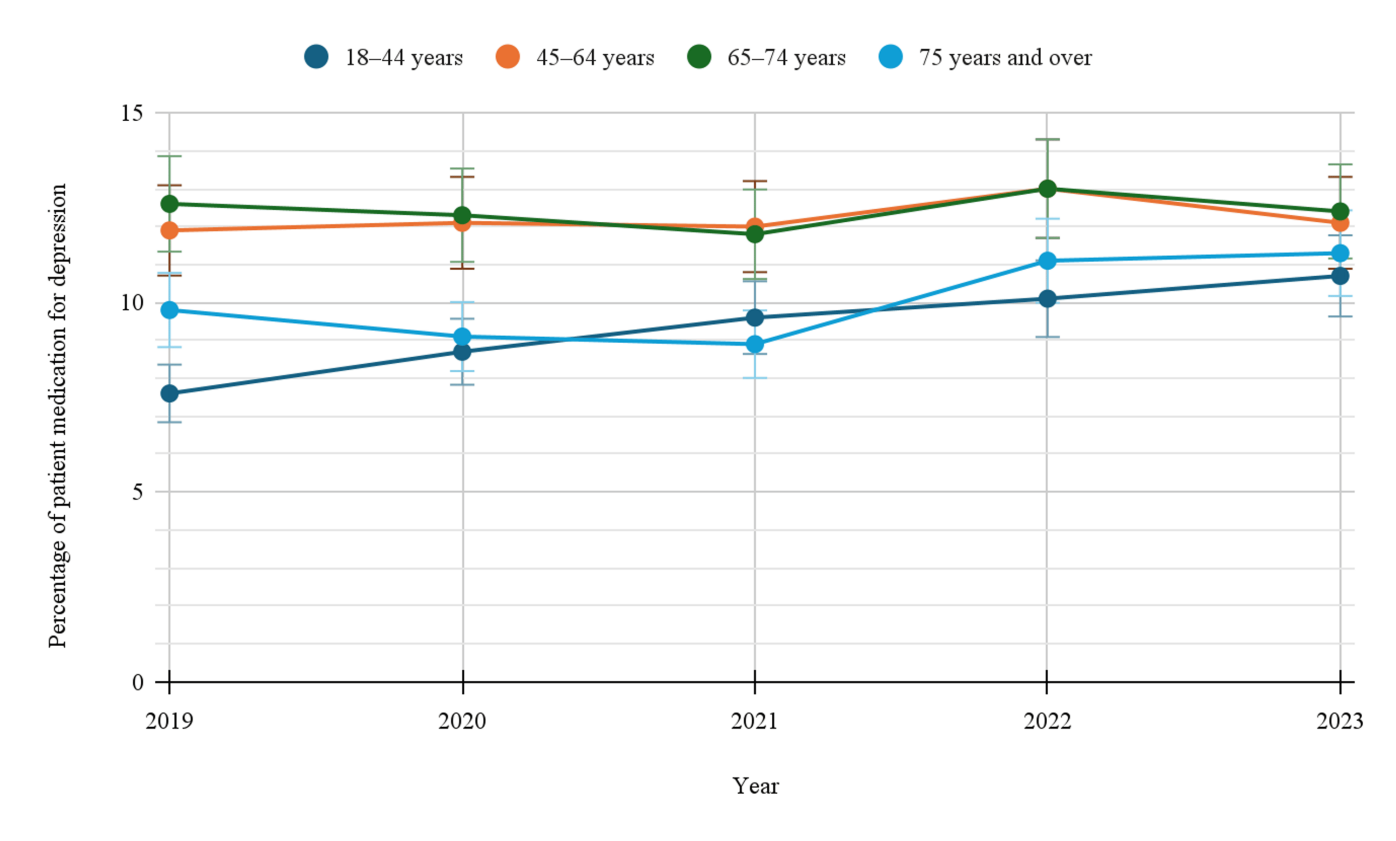
Trends in depression-related prescription medication use by age groups

When analyzed by age groups, antidepressant usage demonstrated age-related variations, with prevalence generally increasing with age, particularly up to the 65-74 years’ category. Among individuals aged 18-44 years, usage rose from 7.6% (95%CI: 7.1-8.2) in 2019 to 10.7% (95%CI: 10.1-11.4) in 2023. The 45-64 age group consistently reported higher prevalence, starting at 11.9% (95%CI: 11.1-12.7) in 2019 and reaching a peak of 13.0% (95%CI: 12.2-13.8) in 2022, before slightly declining to 12.1% (95%CI: 11.4-12.9) in 2023. Those aged 65-74 years followed a similar trajectory, with usage ranging between 11.8% (95%CI: 10.8-12.9) and 13.0% (95%CI: 12.0-14.1). Interestingly, individuals aged 75 years and older displayed a lower prevalence compared to the two preceding age brackets, beginning at 9.8% (95%CI: 8.7-11.0) in 2019 and increasing to 11.3% (95%CI: 10.1-12.6) by 2023 (Table 1). The different age groups category showed a statistically significant trend with a p-value of <0.05, suggesting meaningful changes in this subgroup over time. Figure 2 indicates the trends in depression-related prescription medication use by age groups.

Based on veteran status

Veteran status showed only minimal differences in antidepressant usage (Table 1). In 2019, veterans had a prevalence of 10.1% (95%CI: 8.9-11.5), which increased modestly to 11.0% (95%CI: 9.6-12.6) by 2023. Non-veterans mirrored the general population, beginning at 9.8% (95%CI: 9.4-10.3) in 2019 and rising to 11.5% (95%CI: 11.0-12.0) in 2023. A statistically significant difference was found based on veteran status, with a p-value of 0.0345. 

Based on race/ethnicity

**Figure 3:**
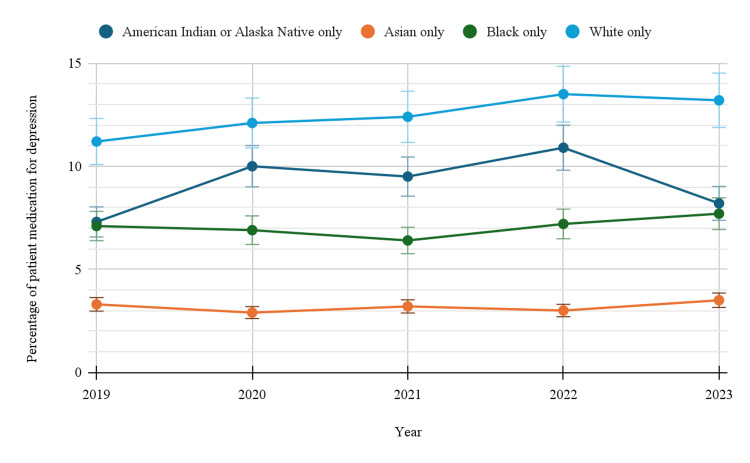
Trends in depression-related prescription medication use by race/ethnicity

Racial disparities in antidepressant usage were also evident. White-only individuals consistently reported the highest rates, increasing from 11.2% (95%CI: 10.7-11.7) in 2019 to 13.2% (95%CI: 12.7-13.8) in 2023. Black-only individuals reported much lower usage, with slight fluctuations from 7.1% (95%CI: 6.2-8.2) in 2019 to 7.7% (95%CI: 6.6-8.8) in 2023. Asian-only individuals had the lowest rates across all years, remaining relatively stable around 3%, ending at 3.5% (95% CI: 2.5-4.6) in 2023. American Indian or Alaska Native individuals showed higher variability, likely due to smaller sample sizes, with usage ranging from 7.3% (95% CI: 3.7-12.6) in 2019 to 8.2% (95%CI: 5.3-12.1) in 2023 (Table 1). The analysis by race showed that individuals had a statistically significant trend over time, with a p-value of <0.05. Figure 3 below indicates the trends in depression-related prescription medication use by race/ethnicity.

Based on social vulnerability

**Figure 4:**
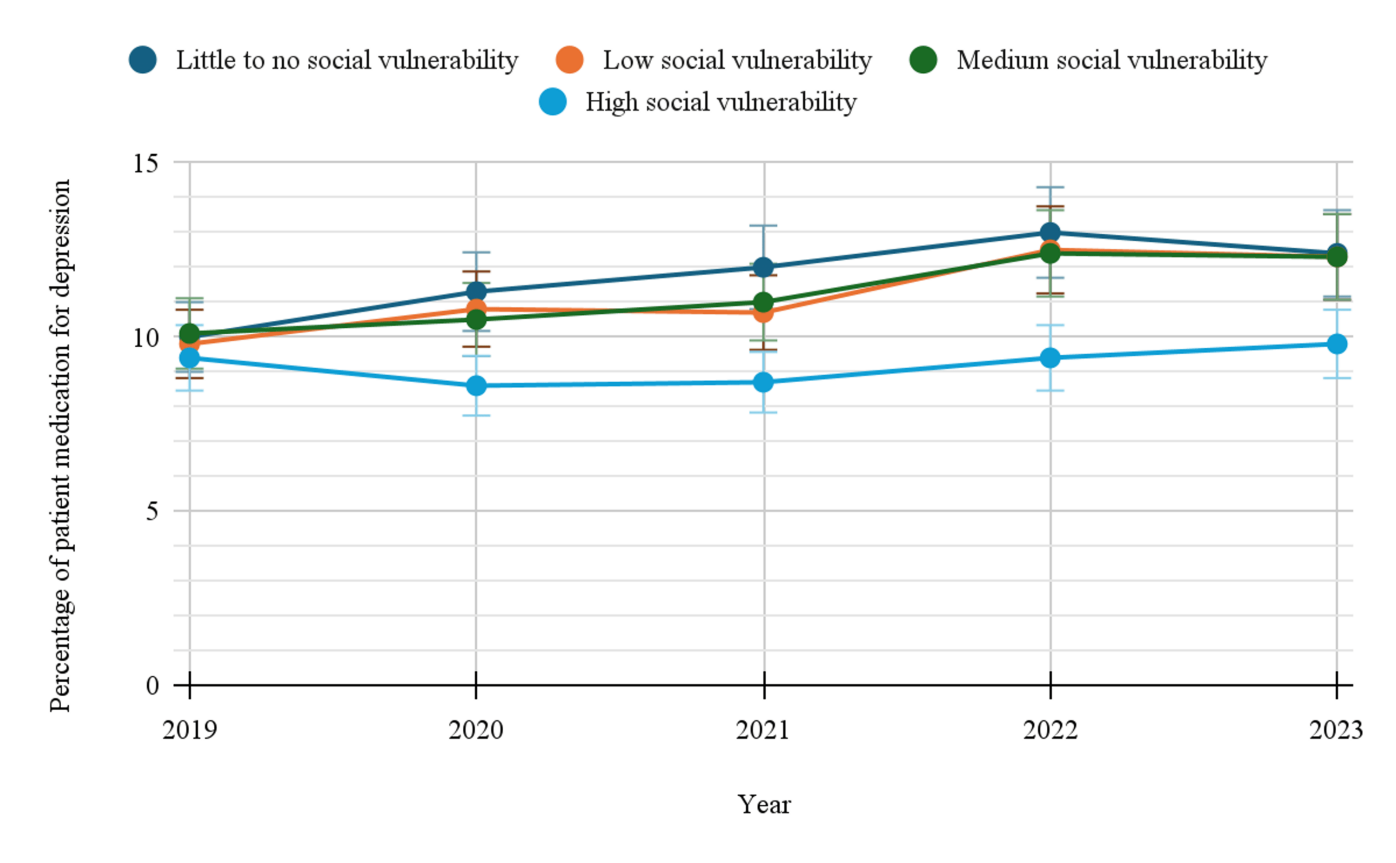
Trends in depression-related prescription medication use by social vulnerability

Social vulnerability appeared to influence antidepressant usage, with a general trend of higher prevalence among those with lower vulnerability. Those with little to no social vulnerability saw an increase from 10.0% (95%CI: 9.0-11.0) in 2019 to 12.4% (95%CI: 11.2-13.6) in 2023 (Table 1). Similarly, individuals with low and medium social vulnerability recorded increasing trends. In contrast, those with high social vulnerability showed lower and less variable usage, with prevalence moving modestly from 9.4% (95%CI: 8.5-10.3) to 9.8% (95%CI: 9.1-10.5). Among individuals with different levels of social vulnerability, there was a statistically significant trend across the years, with a p-value of 0.004465. Figure 4 below indicates the trends in depression-related prescription medication use by social vulnerability.

Based on disability

One of the most striking disparities emerged when comparing individuals with and without disabilities. Antidepressant use among those with disabilities was exceedingly high, beginning at 27.7% (95%CI: 25.8-29.6) in 2019 and remaining elevated at 28.2% (95%CI: 26.3-30.1) in 2023. In contrast, individuals without disabilities reported substantially lower rates, increasing only slightly from 8.1% (95%CI: 7.7-8.5) in 2019 to 9.7% (95%CI: 9.3-10.2) in 2023 (Table 1). The analysis indicated a statistically significant trend for individuals without a disability, with a p-value of <0.05. 

Based on the federal poverty level

**Figure 5:**
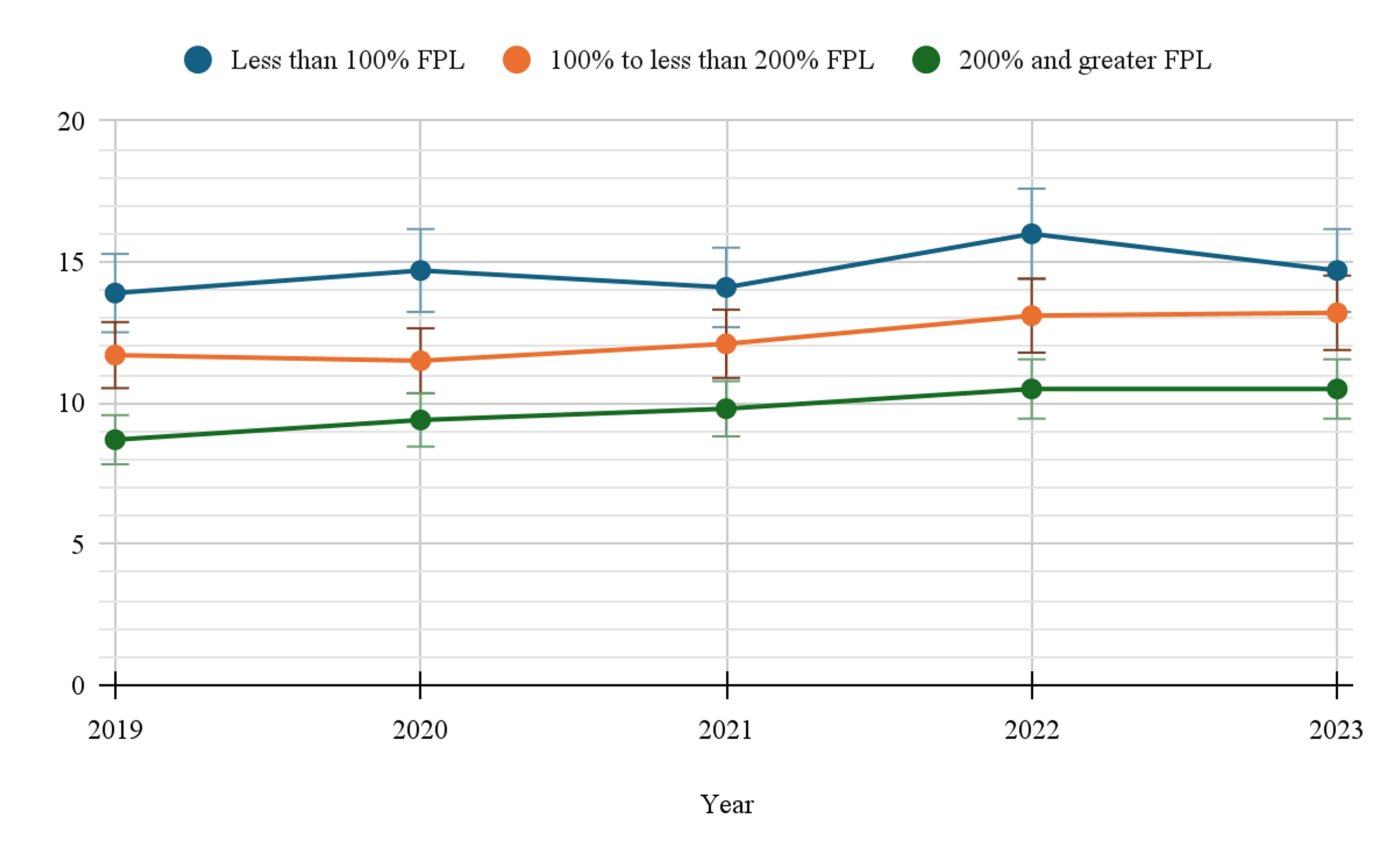
Trends in depression-related prescription medication use by poverty level FPL: Federal poverty level

Socioeconomic status, represented by the federal poverty level (FPL), also played a significant role in antidepressant use. Individuals below 100% FPL consistently showed the highest prevalence, starting at 13.9% (95%CI: 12.4-15.5) in 2019 and ending at 14.7% (95%CI: 13.3-16.3) in 2023. Those in the 100% to <200% FPL category followed closely behind, with use increasing from 11.7% (95%CI: 10.7-12.8) to 13.2% (95%CI: 12.0-14.4). Meanwhile, individuals at 200% or greater FPL had consistently lower usage, rising modestly from 8.7% (95%CI: 8.3-9.1) to 10.5% (95%CI: 10.0-11.0) (Table 1). Statistically significant changes were observed for the group based on FPL, with a p-value of <0.05. Figure 5 below indicates the trends in depression-related prescription medication use by poverty level.

Based on insurance

Insurance coverage type was another key determinant of antidepressant use. Individuals with private insurance had the lowest usage among the insured, increasing from 8.4% (95%CI: 7.9-8.9) in 2019 to 10.6% (95%CI: 10.0-11.3) in 2023. In contrast, those with Medicaid or other public coverage consistently reported higher use, peaking at 18.1% (95%CI: 16.4-19.8) in 2022. The highest rates were observed among individuals with other coverage types (e.g., Medicare or military), with usage reaching 21.3% (95% CI: 18.1-24.7) in 2023. Uninsured individuals, on the other hand, showed the lowest usage overall, declining from 4.7% (95% CI: 3.8-5.6) in 2019 to 4.0% (95% CI: 3.1-5.0) in 2023 (Table 1). Among insurance categories, there were statistically significant trends over time, as indicated by a p-value of <0.05.

## Discussion

This study analyzed the trends in antidepressant medication use in the United States from 2019 to 2023, using data from the NHIS. The findings highlight important patterns in the use of antidepressants across various demographic groups, including gender, age, veteran status, race, social vulnerability, disability, poverty level, and insurance coverage. Our results are consistent with previous studies, but also provide a detailed view of disparities in antidepressant medication use, particularly among vulnerable populations. This discussion will compare the results of this study with existing literature, focusing on trends in antidepressant use, gender disparities, age-related differences, and the influence of social factors such as disability and poverty.

The data from the NHIS revealed a steady increase in antidepressant use from 2019 to 2023, which aligns with national trends observed in previous studies. The overall prevalence of antidepressant use in this study increased from 9.8% in 2019 to 11.4% in 2023, showing a gradual upward trajectory. This increase mirrors the findings of other studies, including a similar analysis of antidepressant use over a longer period. For instance, the study by Mojtabai et al. (2017) found that antidepressant use in the U.S. increased from 7.7% in 2005 to 9.2% in 2014. The growth in antidepressant usage can likely be attributed to better recognition of mental health conditions, reduced stigma, and increased access to treatment options [13].

A study by NCHS on trends in antidepressant prescriptions in the U.S. found a similarly rising trend, particularly among women. Our data also indicate a significant increase in antidepressant use over the study period, with a noticeable difference in gender-based usage [14]. Gender disparities in antidepressant use are well-documented in the literature, and our study supports these findings. In our study, women consistently had higher rates of antidepressant use compared to men, with usage rising from 13.3% in 2019 to 15.3% in 2023, compared to 6.1% in 2019 and 7.3% in 2023 among men. This gender gap is consistent with previous studies. A study indicated that women are more likely to be prescribed antidepressants than men, with prevalence rates around 18% for women compared to 8% for men in the United States [15].

The higher prevalence of antidepressant use in women could be attributed to several factors. Research has shown that women are more likely to seek help for mental health issues and are more likely to be diagnosed with depression than men (Kuehner, 2017) [16]. Additionally, biological and hormonal factors, such as fluctuations in estrogen and progesterone levels, may contribute to the higher rates of depression observed in women, which could, in turn, explain the higher use of antidepressants (Kundakovic and Rocks, 2022) [17].

Antidepressant use also varied significantly across age groups. Younger adults aged 18-44 years saw a gradual increase in antidepressant use from 7.6% in 2019 to 10.7% in 2023, while older adults, particularly those aged 45-64 years and 65-74 years, had higher rates of use. The highest usage was observed in the 65-74 age group, with prevalence peaking at 13.0% in 2022. These results are consistent with previous research that shows higher antidepressant usage among older adults. A study by Haigh et al. (2018) reported that older adults have higher rates of depression and are more likely to receive treatment for depression, including antidepressants [18].

Interestingly, the group aged 75 years and over showed a lower, but still noticeable, increase in antidepressant use, from 9.8% in 2019 to 11.3% in 2023. This finding is consistent with previous studies that suggest older adults may face barriers to receiving mental health treatment, such as underdiagnosis, age-related stigma, and concerns about polypharmacy (Wuthrich and Frei, 2015) [19]. The slight increase in antidepressant use in this group could reflect an improvement in mental health awareness among elderly populations or better access to healthcare in recent years [19].

Veteran status was another important factor influencing antidepressant use. While veterans had slightly higher antidepressant use (peaking at 11.0% in 2023) compared to non-veterans (11.5%), the difference was not substantial. Clinical relevance is modest, suggesting VA initiatives may have achieved parity in antidepressant access [20]. Previous studies have shown that veterans are more likely to suffer from mental health conditions, including depression, due to their experiences in military service. However, despite this increased risk, mental health treatment among veterans may be hindered by barriers such as the availability of services and the stigma associated with seeking help [20]. The Department of Veterans Affairs (VA) has made significant efforts to improve mental health care for veterans, which may explain the relatively high usage of antidepressants in this group [20].

Racial disparities in antidepressant use have been well-documented, and our study further supports this. White individuals had the highest rates of antidepressant use, with usage rising from 11.2% in 2019 to 13.2% in 2023. Black and Asian individuals had lower rates, with Black individuals seeing a slight increase in usage from 7.1% in 2019 to 7.7% in 2023, and Asian individuals showing only marginal fluctuations. These disparities are consistent with other studies that show lower rates of antidepressant use among racial minorities, particularly African Americans and Asian Americans [21].

Racial disparities in mental health care can be attributed to various factors, including socioeconomic status, cultural attitudes toward mental health, and differences in healthcare access. Minority populations may face significant barriers to mental health treatment, including a lack of access to mental health providers, cultural stigma, and distrust of the healthcare system. Addressing these disparities requires targeted interventions to increase mental health awareness and improve access to care for underserved populations [21].

Socioeconomic factors, including poverty and insurance coverage, also played a significant role in antidepressant use. Individuals living below the FPL (Federal Poverty Level) consistently showed higher rates of antidepressant use, peaking at 14.7% in 2023. This finding is consistent with research that suggests individuals with lower socioeconomic status are more likely to experience mental health issues due to factors such as financial stress, limited access to healthcare, and lack of social support [22].

Insurance coverage was another important determinant of antidepressant use. Those with private insurance had the lowest prevalence, while individuals with Medicaid or other public insurance showed higher rates. This reflects the importance of healthcare coverage in facilitating access to antidepressant medications. Previous research has found that individuals with public insurance are more likely to use mental health services, including prescription medications [23,24].

The most striking finding in our study was the significantly higher rates of antidepressant use among individuals with disabilities. The prevalence in this group was more than three times higher than in those without disabilities, peaking at 29.6% in 2022. This result is consistent with previous studies that have found higher rates of depression and antidepressant use among individuals with disabilities [25]. Individuals with disabilities are at a greater risk for mental health issues due to factors such as physical limitations, social isolation, and discrimination. Mental health services must be tailored to the needs of this population to ensure they receive adequate care [26,27].

Strengths and limitations

This study provides valuable insights into the trends of antidepressant medication use across diverse demographic groups from 2019 to 2023, using a robust dataset from the National Health Interview Survey (NHIS). One of the main strengths of this study is its large, nationally representative sample, which ensures generalizability of the findings to the broader U.S. population. The use of longitudinal data allows for an examination of trends over five years, providing a comprehensive view of how antidepressant use has evolved across different socio-demographic groups. Additionally, the study highlights critical disparities in medication use based on gender, age, race, and socioeconomic factors, offering valuable implications for targeted mental health policies.

However, there are several limitations. First, the data rely on self-reported information, which could introduce biases such as recall or social desirability bias, affecting the accuracy of antidepressant use reporting. Second, the study does not account for the specific types or dosages of antidepressants prescribed, nor does it capture other mental health treatments, which limits a deeper understanding of treatment patterns. Third, the NHIS does not provide information on the severity of depressive symptoms, presence of comorbid psychiatric or chronic health conditions, or clinical diagnoses beyond symptom screening, constraining insight into how these factors influence medication use. Despite these limitations, the study provides critical insights into trends and disparities in antidepressant medication use.

These findings underscore the need for clinicians and policymakers to ensure equitable access to mental health services, particularly for socially vulnerable and disabled populations. Future research should integrate electronic health record data or clinical registries to capture diagnostic severity, treatment adherence, and comorbidity profiles, enabling more nuanced analyses of antidepressant utilization and outcomes. Such efforts will facilitate tailored interventions to improve treatment efficacy and mental health outcomes across diverse populations.

## Conclusions

In conclusion, the analysis of antidepressant medication use from 2019 to 2023 reveals significant trends and disparities across various demographic factors, including gender, age, race, veteran status, and social vulnerability. Overall, antidepressant use increased steadily over the study period, with higher rates observed in females, older adults, and individuals with disabilities. Gender differences were particularly pronounced, with women consistently showing higher usage than men. Racial disparities were also evident, with white individuals exhibiting the highest prevalence of antidepressant use, while minority groups, such as Black and Asian populations, had lower rates. Additionally, socioeconomic factors like poverty level and insurance coverage influenced antidepressant use, with those in lower income brackets and those covered by Medicaid or other public insurance showing higher usage rates. These findings highlight the importance of addressing the underlying social and demographic factors contributing to disparities in mental health treatment and ensuring better access to antidepressant medications for underserved populations. Policymakers should implement targeted outreach and expanded tele-mental health initiatives to improve equitable access to depression care.
